# Novel synthetic procedures for C2 substituted imidazoquinolines as ligands for the α/β-interface of the GABAA-receptor

**DOI:** 10.1007/s00706-022-02988-8

**Published:** 2022-10-29

**Authors:** Markus Draskovits, Daniele Catorci, Laurin Wimmer, Sabah Rehman, David Chan Bodin Siebert, Margot Ernst, Michael Schnürch, Marko D. Mihovilovic

**Affiliations:** 1 Institute of Applied Synthetic Chemistry, TU Wien, Getreidemarkt 9/163, 1060 Vienna, Austria; 2 Center for Brain Research, Medical University of Vienna, Spitalgasse 4, 1090 Vienna, Austria

**Keywords:** GABA-induced current modulation, Direct C–H arylation, Heterocyclic chemistry, Medicinal chemistry

## Abstract

A series of substituted imidazoquinolines, a structurally related chemotype to pyrazoloquinolinones, a well-known class of GABA_A_ ligands, was prepared via two synthetic procedures and the efficiency of these procedures were compared. One method relies on classical heterocyclic synthesis, the other one aims at late-stage decoration of a truncated scaffold via direct C–H functionalization. A pharmacological evaluation disclosed that one of the synthesized derivatives showed interesting activity on a α1β3 containing receptor subtype.

**Graphical abstract:**

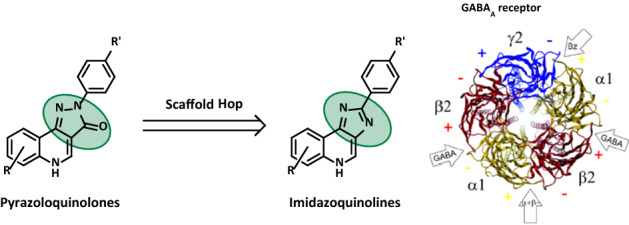

**Supplementary Information:**

The online version contains supplementary material available at 10.1007/s00706-022-02988-8.

## Introduction

The endogenous neurotransmitter, γ-aminobutyric acid (GABA), plays a major role in neurotransmission in the mammalian brain, where it binds to ligand-gated ion channel type A receptors (GABA_A_) [1, 2]. These pentameric receptors are composed of different subunits (α1-6, β1-3, γ1-3, δ, ε, π, θ, ρ1–3) and the specific assemblies thereof (receptor subtype) determine their pharmacological effects [3]. Upon GABA binding, an anion flux is induced, which can be affected by a variety of allosteric modulators. This is of clinical importance in various ways. For example, it has been reported that sedative and anxiolytic effects are elicited by allosteric modulators (such as benzodiazepines), depending on the particular receptor subtype [4].

Allosteric modulators which use a large number of allosteric sites [5] are in widespread use as anaesthetics, anticonvulsants, sedatives, and tranquilizers [6, 7]. Moreover, they are known to bind to the α + /γ- interfaces of the pentameric receptors [8]. Nevertheless, the treatment with these drugs is accompanied by several severe side effects, such as addictive behaviour and withdrawal syndromes [9]. Hence, there is an urgent need for new pharmaceutical ligands with a reduced side effect profile.

Another class of exogenous ligands for the GABA_A_ receptors are pyrazoloquinolinones which showed interesting modulatory activities via a different binding site, namely the α + /β- interface [10]. However, these compounds still possess a promiscuous binding profile [2] and poor solubility in most polar solvents. Thus, they are investigated to become pharmacological tool compounds by variation of their substitution pattern [11–16].

**Fig. 1:**
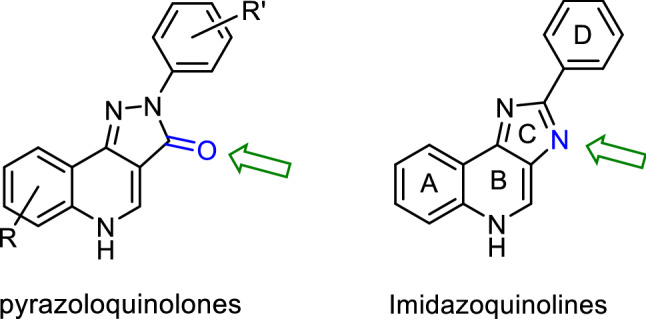
The change of the hydrogen bond donor entity is highlighted in blue and indicated by the green arrow

In line with the concept of pharmacophore modelling, the class of imidazoquinolines are a closely related chemotype to the pyrazoloquinolinones and differs mainly in the change of one hydrogen bond acceptor entity (Fig. 1). Therefore, they represent a promising class of compounds in terms of GABA_A_ receptor activity [17]. Since synthetic routes towards this peculiar tricyclic scaffold are scarce [18–20], we wanted to investigate an alternative method to gain flexibility in synthesis. High functional group tolerance was the main prerequisite for a newly developed method. Moreover, we aimed to investigate the modulatory activity of the newly synthesized compounds at a GABA_A_ receptor subtype.

## Results and discussion

To allow the introduction of various substituents on ring D, a retrosynthetic analysis of lead structure **I** suggested two main building blocks: the diamino quinoline **II** and benzaldehyde or benzoic acid **III** (Scheme 1). This route allows late-stage modifications of substituents R^1^ on ring D via the use of different benzaldehyde or benzoic acid derivatives. However, the diamino quinoline **II** has to be synthesized for each substituent R^2^.

**Figure:**
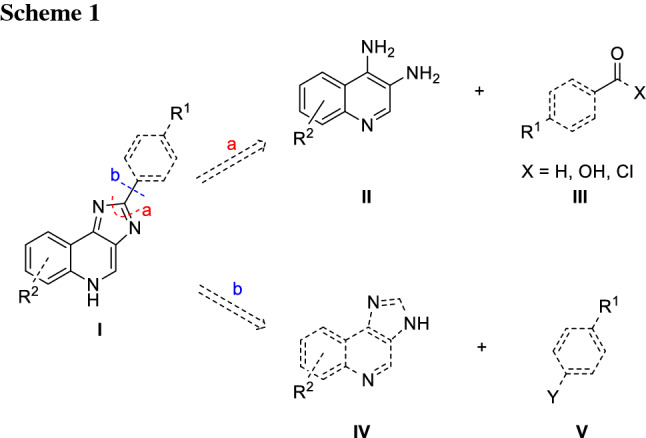

The alternative retrosynthetic cut b (Scheme 1) leads to **IV** and an appropriated substituted benzene **V**. Following this pathway, a direct arylation in position 2 can be envisioned taking advantage of transition metal catalysed C-H functionalization. In addition, in this case the corresponding building block **IV** would need to be prepared for each substituent R^2^ separately. Hence the difference lies in the final introduction of the aryl-moitey at C2. Additionally, it has to be kept in mind, that the C–H coupling at C2 might be in competition with N-arylation. To determine, which method would be the overall more efficient one, both approaches were investigated.

For both approaches, diaminoquinolines have to be prepared as a crucial intermediate. Synthetic routes towards these compounds have been published previously and were also established partially in our group [12].

The aniline starting materials **1a**–**1d** determine the substitution pattern on ring A in the final molecule. Building up on our findings from the study on pyrazoloquinolinones we decided to use the unsubstituted aniline **1a**, two halogenated anilines **1b** and **1c** and anisidine **1d** to introduce a methoxy substituent [12]. The first step of the synthesis was the condensation of the anilines **1a**–**1d** to the quinolones **3a**–**3d**. This was done in a two-step process. In the first step, the anilines **1a**–**1d** reacted with diethylmalonate in a Gould-Jacobs reaction [21] to form the corresponding enamines **2a**–**2d** in excellent yields (90%—quantitative). Then, after a solvent exchange to a higher boiling solvent, the ring closure to quinolones **3a**–**3d** was performed. In this step, high temperatures were required (> 150 °C) since intermediately aromaticity is lost. Not surprisingly, such high temperatures led to the formation of a significant amount of (unidentified) side products. Nevertheless, the desired carboxylated quinolones **3a**–**3d** were obtained in acceptable yields (31–61%) and good purity, as they were hardly soluble in any organic solvent and could be easily purified by trituration. The decarboxylation of the ester group was achieved also in a two-step process: first, the ester was hydrolysed and after the carboxylic acid was isolated by precipitation, it was thermally decarboxylated to yield the quinolones **4a**–**4d**, again by using Ph_2_O as a high-boiling solvent (Scheme 2).

**Figure:**
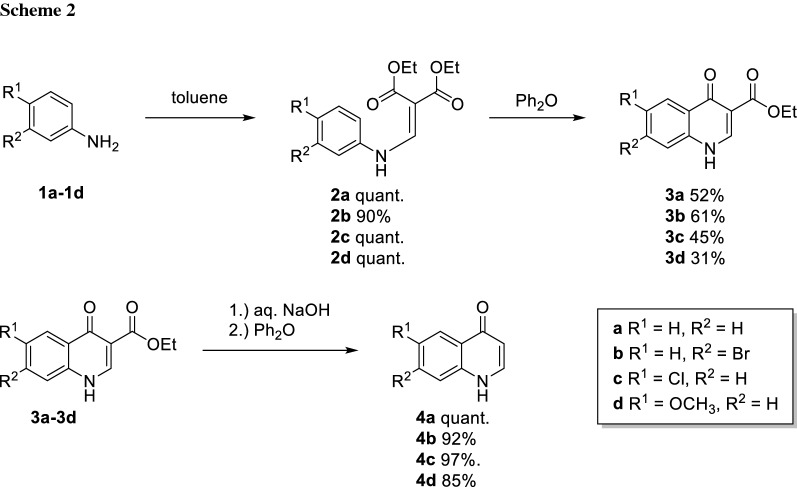

Then, we introduced the nitro group in position 3 which was accomplished by standard nitration conditions using a mixture of nitric and acetic acid which gave the 3-nitroquinolones **5a**–**5d** in good yields. Subsequently, chlorination was performed using POCl_3_, yielding the chlorinated nitroquinolines **6a**–**6d** (Scheme 3).

**Figure:**
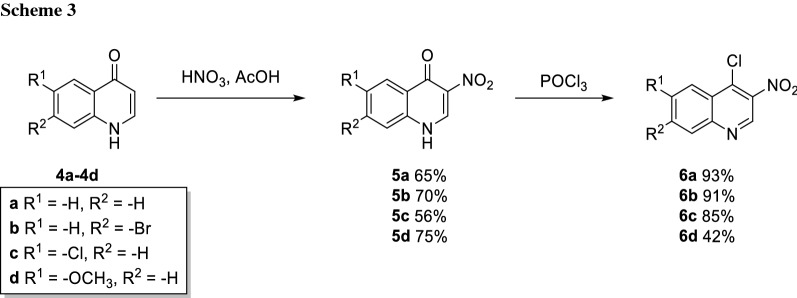

The final steps towards the diamino quinolines now differed for halogen-bearing derivatives **6b** and **6c**, as milder reduction conditions had to be employed to avoid dehalogenation. Therefore, the amino group on position 4 was introduced directly using aq. ammonia in 1,4-dioxane, which gave the amino-nitro quinolines **7a**–**7c** in quantitative yields. Reduction of the nitro group was carried out under modified Béchamp conditions [22] using iron and NH_4_Cl to give diamino quinolines **8a**–**8c** (Scheme 4). If other reduction conditions, like palladium on charcoal under H_2_ atmosphere were employed, substantial dehalogenation was observed.

**Figure:**
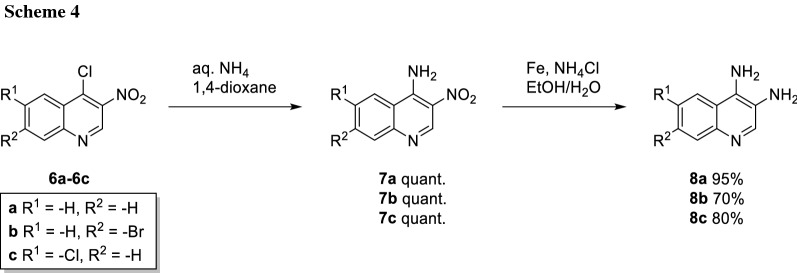

In cases where dehalogenation was not an issue, a route via azide **7d** can be applied. Subsequent reduction using palladium on charcoal under a hydrogen atmosphere afforded the desired diamino quinoline **8d** (Scheme 5).

**Figure:**
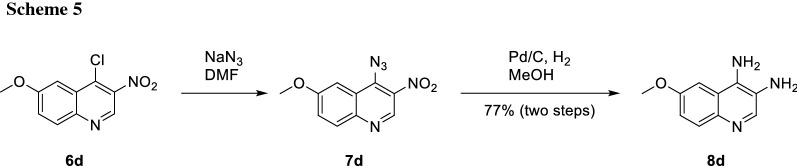

The diamino quinolines **8a**–**8d** now enabled the syntheses of the desired imidazoquinolines. Initially, compounds **9a**–**9d** were prepared to test the direct arylation approach. Cyclization of **8a**–**8d** with formic acid led to the target compounds in good to excellent yields (Scheme 6, 57–98%). Optimization of the direct arylation was carried out using **9a** as substrate, as the simplest derivative of this series. First, we employed a protocol established by Bellina et al. for imidazoles, azoles, indoles, and benzimidazoles [23]. There, the C–H activation is carried out by a palladium-copper complex under ligand- and base-free conditions in DMF. When we applied the same conditions on **9a**, the direct arylation towards the desired product **10a** occurred, but only in 15% yield after a reaction time of 48 h (Table 1, entries 1 and 2). In this first experiment, we observed two main issues which decreased the formation of product. Most importantly, N-arylation of the nitrogen in position three indeed took place and led to significant side product formation. Furthermore, the solubility of the starting material **9a** was low and precipitation occurred during the reaction.

**Figure:**
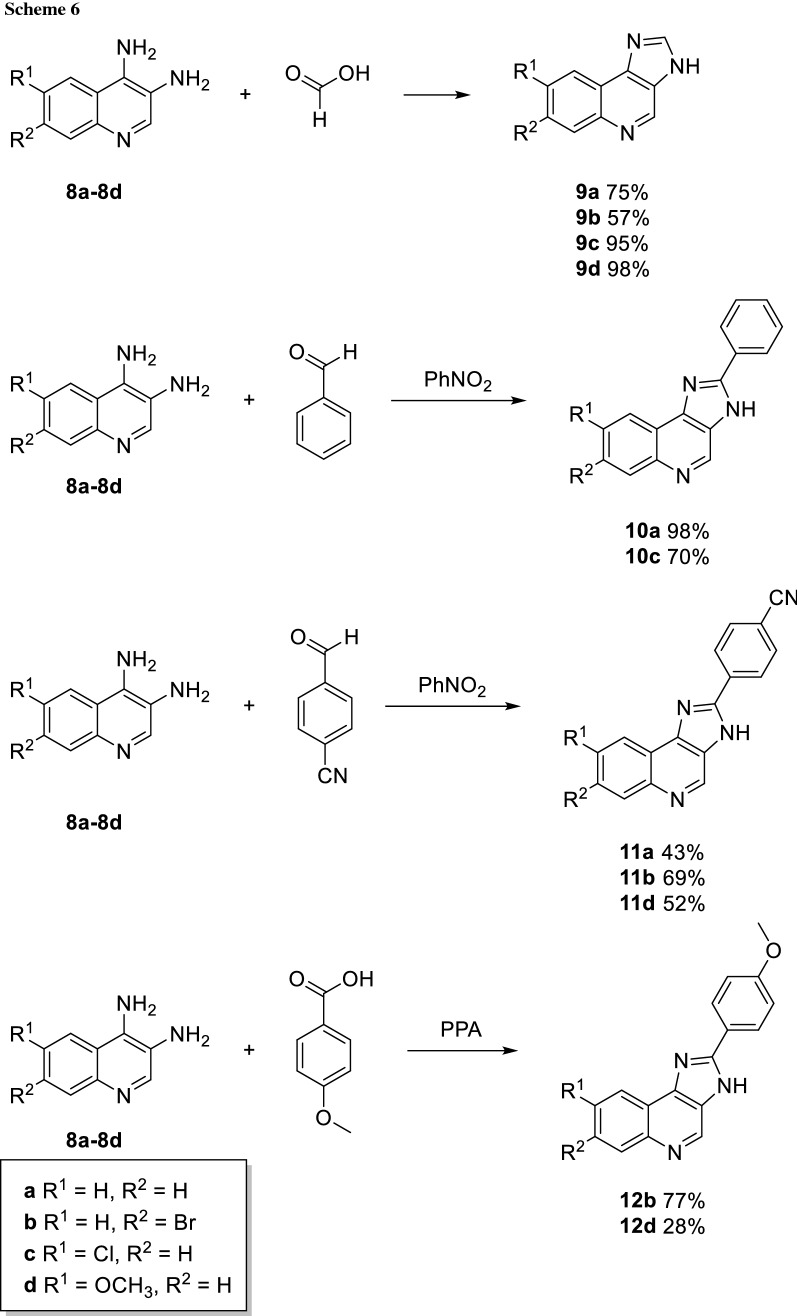

**Table 1:** Conditions for the optimization of the CH activation


Entry	CuI	Base	Time (h)	Yield
1	2 equiv	–	12	Traces
2	2 equiv	–	48	15%
3	2 equiv	DBU	48	–
4	2 equiv	Cs_2_CO_3_	48	–
5	10 mol%	–	48	20%
6	0.6 equiv	–	48	34%

Reactions were carried out using Ar-I (2 equiv.), Pd(OAc)_2_ (5 mol%), DMF, and 140 °C

Therefore, the catalytic system by displacement of palladium with a different transition metal species (Rh and Ni catalysts, see SI for details). In addition, the influence of different bases as well as ligands and additives was investigated (Table 1, entries 3 and 4, and SI). As this only had limited success, we built upon our initial successful transformation using Pd(OAc)_2_ and CuI in DMF and focused on finding conditions which prevent the precipitation of starting material. This might be caused by the formation of a copper salt, which has been reported previously for similar heterocyclic systems [24]. Therefore, we tried to reduce the amount of the copper source to a minimum which still allows the transformation. We found that a decreased amount of copper additive (0.6 equiv.) under prolonged reaction times (96 h) led to an increased isolated yield of 34% of the desired **10a** (Table 1, entry 6). Further attempts to increase the yield by *N*-protection to suppress side product formation and to increase solubility failed (see SI).

Consequently, we switched to a more classical cyclization approach using either carboxylic acids or aldehydes as reaction partners. Benzaldehyde was used in combination with nitrophenol (as solvent and oxidant) to obtain the desired imidazoquinolines **10a** and **10c** [25]. The same conditions using now *p*-formylbenzonitrile could be applied to introduce a cyano group on ring D in the final product. This afforded the desired products **11a**, **11b**, and **11d** in good yields (Scheme 6). However, the same methodology led only to the intermediary formed imine with *p-*methoxybenzaldehyde. Thus, we decided to use the more stable carboxylic acid under harsher conditions. Polyphosphoric acid, a strong acidic and hygroscopic reagent, which additionally binds the formed water allowed the synthesis of the methoxy substituted imidazoquinolines **12b** and **12d**.

**Fig. 2:**
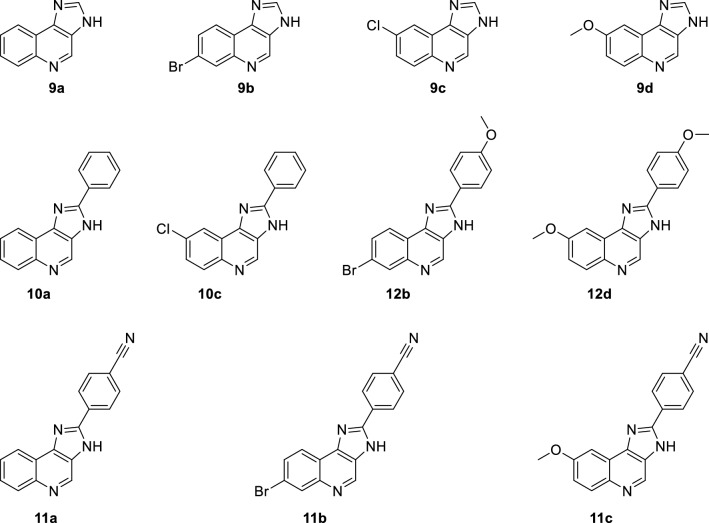
Library of synthesized imidazoquinolines

Overall, we synthesized a library of 11 imidazoquinolines which we aimed to investigate their activity as allosteric modulators in GABA_A_ receptors (Fig. 2).

The modulatory activity was investigated by testing the ligands in recombinant α1β3 GABA_A_ receptors. The receptors were expressed in *X. laevis* oocytes and the compounds were screened at 1 µM and 10 µM at 3–5% (EC_3-5_) of the maximum GABA elicited current (see methods).

**Fig. 3:**
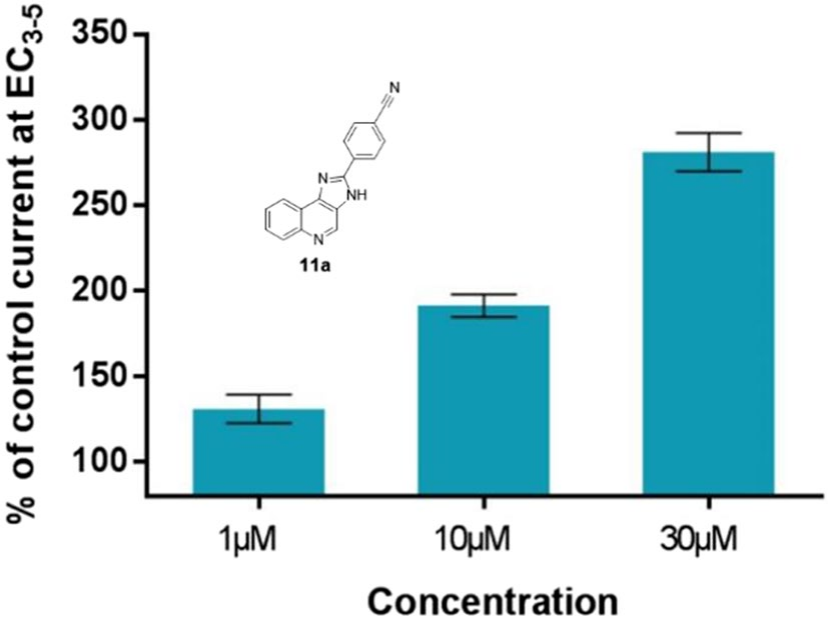
Modulatory activity of compound **11a** in α1β3. Compound **11a** was tested in the GABA_A_ receptor α1β3 (*n* = 4) at 1 µM, 10 µM, and 30 µM at 3–5% of maximum elicited GABA current

Compounds **11a**, **11b**, **11d** (cyano substituted D ring) and **12b**, **12d** (methoxy substituted D ring) were tested to compare their activity with similarly substituted pyrazoloquinolinones [7, 8]. Interestingly, compound **11a** displayed a significant modulatory activity (Fig. 3), while the other compounds were not sufficiently active to further characterize in this receptor subtype. Thus, compound **11a** represents to the best of our knowledge the first imidazoquinolone which significantly modulates GABA_A_ receptors independently of the presence of a benzodiazepine binding site.

## Conclusion

We were able to establish a versatile synthetic route towards the class of imidazoquinolines, which already showed interesting activity on the tested GABA_A_ receptor subtype. Compound **11a** gave the most promising results and is the first example of a positive allosteric modulator of its class. The synthetic route was adapted in a way to allow the introduction of a large number of substituents, especially via the first shown CH activation of an imidazoquinoline towards 3-arylimidazoquinolines. Additionally, it is important to note that the imidazoquinoline products **10**, **11**, and **12** displayed increased solubility as compared to the corresponding pyrazoloquinolinones. Future research will be directed towards further improving the activity profile and establish the imidazoquinolones as subtype-selective GABA_A_ receptor ligands.

## Experimental

All starting materials and reagents were purchased from commercial sources and used without further purification. Reactions were monitored by TLC on silica gel 60 F254 plates. Normal-phase column chromatography was performed on silica gel 60 (230–400 mesh). NMR spectra were recorded at 297 K in the solvent indicated, with 200, 400, and 600 MHz instruments, respectively, employing standard software provided by the manufacturer. ^1^H NMR and ^13^C NMR spectra were referenced to tetramethylsilane (TMS, *δ* = 0) by calibration with the residual organic solvent signals [26]. Accurate mass analysis (2 ppm mass accuracy) was carried out from 10 to 100 mg/dm^3^ solutions via LC-TOFMS measurements using an autosampler, an HPLC system with binary pumps, degasser, and column thermostat and ESI-TOF mass spectrometer. Melting points were determined with a Büchi Melting Point B-545 apparatus with a heating rate of 1 °C/min (70% onset point and 10% clear point) or on a Kofler Block apparatus. All melting points were obtained without additional recrystallization directly after flash column chromatography (FCC) with light petroleum (LP) and EtOAc and subsequent drying in a high vacuum.

### Two-electrode voltage clamp electrophysiology

All steps were performed as reported previously [14]. cDNA vectors were linearized, transcribed and purified to generate mRNAS, which were used for injecting *Xenopus laevis* oocytes. For the microinjection, the RNA of the α1β3 receptor combination was mixed at 1:1 ratio with a final concentration of 56 ng/mm^3^. Oocytes were obtained from commercial or local academic suppliers. Stage 5–6 oocytes with the follicle cell layer around them were roughly dissected with forceps, digested with collagenase (type IA, Sigma, NO, 1 mg/ml ND96 [96 mM NaCl, 2 mM KCl, 1 mM MgCl_2_, 5 mM HEPES; pH 7.5)] at 18 °C shaking at 70 rpm for 20–40 min and gently defolliculated with the aid of a pipette and a platinum loop. For electrophysiological recordings, oocytes were placed on a nylon-grid in a bath of Ca^2+^-containing NDE solution medium [96 mM NaCl, 5 mM HEPES–NaOH (pH 7.5), 2 mM KCl, 1 mM MgCl_2_‧6H_2_O, 1.8 mM CaCl_2_‧2H_2_O]. For current measurements, the oocytes were impaled with two microelectrodes (1–3 MΩ) filled with 2 M KCl. The oocytes were constantly washed by a flow of 6 cm^3^/min NDE that could be switched to NDE containing GABA and/or drugs. Drugs were diluted into NDE from DMSO-solutions resulting in a final concentration of 0.1% DMSO perfusing the oocytes. DMSO was used as blind control. Compounds were co-applied with GABA until a peak response was observed. All recordings were performed at room temperature at a holding potential of − 60 mV using a Dagan TEV-200A two-electrode voltage clamp (Dagan Corporation, Mineapolis, MN). Data were digitized, recorded and measured using an Axon Digidata 1550 low-noise data acquisition system (Axon Instruments, Union City, CA). Data acquisition was done using pCLAMP v.10.5 (Molecular Devices™, Sunnyvale, CA). Data were analysed using GraphPad Prism v.6. and plotted as bar diagrams/bar graphs. Data are given as mean ± SEM from at least three oocytes of two and more oocyte batches.

### General procedure a: synthesis of malonates

According to a modified literature procedure [27], anilines **1a**–**1d** (1.00 equiv.) and diethyl-2-ethoxymethylene malonate (1.00 equiv.) were dissolved in toluene (1.25 cm^3^/mmol). The reaction was heated to reflux, up to 22 h, until full conversion was observed by TLC (LP/EtOAc, 3:1). The reaction mixture was cooled to rt and the solvent was removed under reduced pressure. The oil residue obtained was lyophilized to give the desired products **2a**–**2d** in adequate purity.

### General procedure B: cyclization to quinolones

According to a modified literature procedure [28], malonates **2a**–**2d** (1.00 equiv.) were dissolved in diphenyl ether (2 cm^3^/mmol), the atmosphere changed to argon and the reaction mixture was heated to reflux for one hour. The reaction time did not allow full conversion but disabled the formation of side products. The reaction mixture was cooled to rt and poured into LP to precipitate the desired quinolone which was collected by filtration and washed several times with a mixture of LP/EtOAc (1:1) to obtain the desired products **3a**–**3d**.

### General procedure C: decarboxylation

According to a modified literature procedure [29], quinolone carboxylates **3a**–**3d** (1.00 equiv.) were suspended in 2 N NaOH solution (20 cm^3^/mmol). The reaction mixture was heated to reflux, up to 4 h, until full conversion was observed by TLC (CH_2_Cl_2_/MeOH, 9:1). The reaction mixture was cooled to rt and neutralized with 2 N HCl to precipitate the product. The product was collected by filtration, washed with 200 cm^3^ water and dried. Decarboxylation was performed in Ph_2_O (20 cm^3^/mmol). Carboxylic acid (1.00 equiv.) was suspended and heated to reflux (250 °C) up to 2 h until full conversion was observed by TLC (CH_2_Cl_2_/MeOH, 9:1). The reaction mixture was cooled to rt and poured into LP to precipitate the desired products **4a**–**4d** which were collected by filtration, washed several times with LP and dried*.*

### General procedure D: nitration

According to a modified literature protocol [30], quinolones **4a**–**4d** (1.00 equiv.) were dissolved in AcOH (15 cm^3^/mmol) under heating. Concentrated HNO_3_ (2.2 equiv.) was diluted with AcOH (1:10) and added to the reaction mixture dropwise. The reaction mixture was heated to reflux for up to 4 h until the consumption of starting material was observed by TLC (CH_2_Cl_2_/MeOH, 9:1). The reaction mixture became orange colored. After cooling to rt, the reaction was poured into water, whereupon the product precipitated. The precipitate was collected by filtration and washed with small amounts of EtOH and water and dried to yield the desired products **5a**–**5d**.

### General procedure E: chlorination of nitroquinolones

According to a modified literature protocol [31], nitroquinolones **5a**–**5d** (1.00 equiv.) were dispersed in POCl_3_ (4.00 equiv.). The reaction mixture was heated to reflux for up to 4 h, until full consumption of the starting material was observed by TLC (LP/EtOAc, 3:1). The reaction mixture was poured on a small amount of ice whereupon precipitation occurred and neutralized with sat. aq. NaHCO_3_. The aqueous layer was extracted with CH_2_Cl_2_, washed with brine, dried over Na_2_SO_4_ and the solvent evaporated. The products **6a**-**6d** were purified by FCC (gradient of 5%-15% EtOAc in LP).

### General procedure F: synthesis of nitroquinolin-4-amines

According to a modified literature procedure [32], nitroquinolones **6a**–**6c** (1.00 equiv.) were dissolved in 1,4-dioxane (10 cm^3^/mmol) and aq. NH_4_OH (25%, 10 cm^3^/mmol) was added. The reaction mixture was heated to reflux up to 1 h, until full consumption of starting material was observed by TLC (CH_2_Cl_2_/MeOH, 19:1). After cooling to rt, a precipitate was formed. The solvent was evaporated and the precipitate was dissolved in EtOAc and extracted with water and brine, dried over Na_2_SO_4_ and evaporated to give the desired products **7a**–**7c**.

#### Diethyl 2-[(phenylamino)methylene]malonate (2a)

[33] Applying general procedure A, using aniline (**1a**, 3.92 cm^3^, 43 mmol, 1.00 equiv.) and diethyl(ethoxymethylene)malonate (8.69 cm^3^, 43 mmol, 1.00 equiv.) were dissolved in toluene (54 cm^3^), giving **2a** (11.4 g, 43 mmol, quant.). Light-pink waxy solid; m.p.: 47–49 °C (Ref. [33] 46–48 °C); ^1^H NMR (400 MHz, DMSO-*d*_6_): *δ* = 1.15–1.32 (m, 6H), 4.12 (q, *J* = 7.1 Hz, 2H), 4.21 (q, *J* = 7.1 Hz, 2H), 7.07–7.23 (m, 1H), 7.30–7.50 (m, 4H), 8.41 (d, *J* = 13.9 Hz, 1H), 10.70 (d, *J* = 13.9 Hz, 1H) ppm; ^13^C NMR (101 MHz, DMSO-*d*_6_): *δ* = 14.2, 14.2, 59.4, 59.6, 93.1, 117.5, 124.6, 129.6, 139.4, 151.1, 164.9, 167.3 ppm. Literature reports NMR data solely in CDCl_3_.

#### Diethyl 2-[[(3-bromophenyl)amino]methylene]malonate (2b)

[15] Applying general procedure A, 3-bromoaniline (**1b**, 15.0 g, 87 mmol, 1.00 equiv.) and diethyl(ethoxymethylene)malonate (18.00 g, 87 mmol, 1.00 equiv.) were dissolved in toluene (109 cm^3^), giving **2b** (26.0 g, 78 mmol, 90%). Yellow needles; m.p.: 71–73 °C (Ref. [15] 71–73 °C); NMR spectral data were found to be identical to the ones described in Ref. [15].

#### Diethyl 2-[(4-chlorophenylamino)methylene]malonate (2c)

[28] Applying general procedure A, 4-chloroaniline (**1c**, 5.00 g, 39.2 mmol, 1.00 equiv.) and diethyl(ethoxymethylene)malonate (7.92 cm^3^, 39.2 mmol, 1.00 equiv.) were dissolved in toluene (49 cm^3^), giving **2c** (12.0 g, 39.2 mmol, quant.). Colourless waxy powder; m.p.: 79–81 °C (Ref. [28] 45–46 °C); NMR spectral data were found to be identical with the ones described in Ref. [28].

#### Diethyl 2-[[(4-methoxyphenyl)amino]methylene]malonate (2d)

[27] Applying general procedure A, 4-methoxyaniline (**1d**, 15.00 g, 122 mmol, 1.00 equiv.) and diethyl(ethoxymethylene)malonate (26.4 g, 122 mmol, 1.00 equiv.), giving **2d** (35.7 g, 122 mmol, quant.). Yellow–brown solid; m.p.: 30–32 °C (Ref. [27] 38–40 °C); ^1^H NMR (400 MHz, CDCl_3_): *δ* = 1.31 (t, *J* = 7.1 Hz, 3H), 1.37 (t, *J* = 7.1 Hz, 3H), 3.79 (s, 3H), 4.23 (q, *J* = 7.1 Hz, 2H), 4.29 (q, *J* = 7.1 Hz, 2H), 6.85–6.94 (m, 2H), 7.02–7.11 (m, 2H), 8.43 (d, *J* = 13.8 Hz, 1H), 10.98 (d, *J* = 13.8 Hz, 1H) ppm; ^13^C NMR (101 MHz, CDCl_3_): *δ* = 14.3, 14.4, 55.6, 60.0, 60.2, 92.5, 115.0, 118.8, 132.8, 152.6, 157.2, 165.8, 169.2 ppm.

#### Ethyl 4-oxo-1,4-dihydroquinoline-3-carboxylate (3a)

[33] Applying general procedure B, diethyl 2-[(phenylamino)methylene]malonate (**2a**, 10.00 g, 38 mmol, 1.00 equiv.) was dissolved in Ph_2_O (76 cm^3^) to give **3a** (4.3 g, 20 mmol, 52%). Light brown powder; m.p.: 270–271 °C (Ref. [33] 268–269 °C); ^1^H NMR (400 MHz, DMSO-*d*_6_): *δ* = 1.28 (t, *J* = 7.1 Hz, 3H), 4.21 (q, *J* = 7.1 Hz, 2H), 7.36–7.47 (m, 1H), 7.61 (dd, *J* = 8.4, 1.1 Hz, 1H), 7.65–7.77 (m, 1H), 8.15 (dd, *J* = 8.1, 1.4 Hz, 1H), 8.55 (s, 1H), 12.31 (br s, 1H) ppm; ^13^C NMR (101 MHz, DMSO-*d*_6_): *δ* = 14.3, 59.5, 109.8, 118.7, 124.6, 125.6, 127.2, 132.4 (d), 138.9, 144.8, 164.8, 173.4 ppm.

#### Ethyl 7-bromo-4-oxo-1,4-dihydroquinoline-3-carboxylate (3b)

[34] Applying general procedure B, diethyl 2-[[(3-bromophenyl)amino]methylene]malonate (**2b**, 26.90 g, 79 mmol, 1.00 equiv.) was dissolved in Ph_2_O (160 cm^3^) to give **3b** (14.0 g, 15.1 mmol, 61%). Brown solid; m.p.: 297 °C (decomp.) [Ref. [34] 334–335 °C (decomp.)]; NMR spectral data were found to be identical to the ones described in Ref. [34].

#### Ethyl 6-chloro-4-oxo-1,4-dihydroquinoline-3-carboxylate (3c)

[35] Applying general procedure B, diethyl 2-[(4-chlorophenylamino)methylene]malonate (**2c**, 10.0 g, 33.6 mmol, 1.00 equiv.) was dissolved in Ph_2_O (67 cm^3^) to give **3c** (3.80 g, 15.1 mmol, 45%). Colourless crystals; m.p.: > 300 °C (decomp.) [Ref. [35] > 200 °C (decomp.)]; ^13^C NMR (151 MHz, DMSO-*d*_6_): *δ* = 14.4, 59.8, 110.1, 121.3, 124.7, 128.4, 129.4, 132.6, 137.7, 145.3, 164.6, 172.3 ppm; ^1^H NMR spectral data were found to be identical with the ones described in Ref. [35].

#### Ethyl 6-methoxy-4-oxo-1,4-dihydroquinoline-3-carboxylate (3d)

[36] Applying general procedure B, diethyl 2-[(4-methoxyphenylamino)methylene]malonate (**2d**, 14.30 g, 49 mmol, 1.00 equiv) was dissolved in Ph_2_O (100 cm^3^) to give **3d** (3.8, 15.1 mmol, 31%). Brown solid; m.p.: 258–260 °C (Ref. [36] 274–276 °C); ^13^C NMR (151 MHz, DMSO-*d*_6_): *δ* = 14.4, 55.5, 59.5, 105.5, 108.7, 120.6, 122.2, 128.5, 133.4, 143.7, 156.6, 165.0, 172.9 ppm; ^1^H NMR spectral data were found to be identical with the ones described in Ref. [36].

#### Quinolin-4(1H)-one (4a)

[37] Applying general procedure C, ethyl 4-oxo-1,4-dihydroquinoline-3-carboxylate (**3a**, 2.62 g, 12.10 mmol, 1.00 equiv.) was saponified with 2 N NaOH (240 cm^3^) and decarboxylated with Ph_2_O (240 cm^3^) to give **4a** (1.69 g, 11.6 mmol, quant.). Cream-coloured powder; m.p.: 203–204 °C; NMR spectral data were found to be identical to the ones described in Ref. [36].

#### 7-Bromoquinolin-4(1H)-one (4b)

[38] Applying general procedure C, ethyl 7-bromo-4-oxo-1,4-dihydroquinoline-3-carboxylate (**3b**, 8.00 g, 27.0 mmol, 1.00 equiv.) was saponified with 2 N NaOH (540 cm^3^) and decarboxylated with Ph_2_O (540 cm^3^) to give **4b** (7.20 g, 31.00 mmol, 92%). Colourless solid; m.p.: 345 °C (decomp.) (Ref. [38] 242–244 °C); NMR spectral data were found to be identical to the ones described in Ref. [38].

#### 6-Chloroquinolin-4(1H)-one (4c)

[39] Applying general procedure C, ethyl 6-chloro-4-oxo-1,4-dihydroquinoline-3-carboxylate (**3c**, 3.70 g, 14.7 mmol, 1.00 equiv.) was saponified with 2 N NaOH (300 cm^3^) and decarboxylated with Ph_2_O (300 cm^3^) to give **4c** (2.51 g, 14.00 mmol, 97%). Cream coloured crystals; m.p.: 270–272 °C (Ref. [39] 269–271 °C); ^1^H NMR (400 MHz, DMSO-*d*_6_): *δ* = 6.07 (d, *J* = 7.4 Hz, 1H), 7.59 (d, *J* = 8.9 Hz, 1H), 7.67 (dd, *J* = 8.9, 2.5 Hz, 1H), 7.95 (dd, *J* = 7.8, 3.2 Hz, 1H), 8.01 (d, *J* = 2.4 Hz, 1H), 11.99 (s, 1H) ppm; ^13^C NMR (101 MHz, DMSO-*d*_6_): *δ* = 108.9, 120.8, 123.9, 126.7, 127.8, 131.8, 138.7, 139.9, 175.6 ppm.

#### 6-Methoxyquinolin-4(1H)-one (4d)

[40] Applying general procedure C, ethyl 6-methoxy-4-oxo-1,4-dihydroquinoline-3-carboxylate (**3d**, 3.79 g, 15.0 mmol, 1.00 equiv.) was saponified with 2 N NaOH (300 cm^3^) and decarboxylated with Ph_2_O (300 cm^3^) to give **4d** (2.23 g, 12.7 mmol, 85%). Colourless solid; m.p.: 250–251 °C (Ref. [40] 251–252 °C); ^13^C NMR (101 MHz, DMSO-*d*_6_): *δ* = 55.3, 104.2, 107.5, 120.0, 122.1, 126.8, 134.7, 138.4, 155.4, 176.2 ppm; ^1^H NMR spectral data were found to be identical with the ones described in Ref. [40].

#### 3-Nitroquinolin-4(1H)-one (5a)

[31] Applying general procedure D, quinolin-4(1*H*)-one (**4a**, 2.30 g, 15.8 mmol, 1.00 equiv) was treated with conc. HNO_3_ (1.5 cm^3^) to give **5a** (1.95 g, 10.30 mmol, 65%). Brown solid; m.p.: > 280 °C (decomp.) [Ref. [31] 357–358 °C (decomp.)]; ^1^H NMR (400 MHz, DMSO-*d*_6_): *δ* = 7.47–7.59 (m, 1H), 7.67–7.76 (m, 1H), 7.76–7.86 (m, 1H), 8.26 (dd, *J* = 8.1, 1.5 Hz, 1H), 9.19 (s, 1H), 13.00 (br s, 1H) ppm; ^13^C NMR (101 MHz, DMSO-*d*_6_): *δ* = 119.5(s), 125.8, 126.0, 128.1, 131.0, 133.2, 138.3, 142.4, 167.6 ppm.

#### 7-Bromo-3-nitroquinolin-4(1H)-one (5b)

[41] Applying general procedure D, 7-bromoquinolin-4(1*H*)-one (**4b**, 5.00 g, 22.00 mmol, 1.00 equiv.) was treated with conc. HNO_3_ (2 cm^3^) to give **5b** (4.20 g, 15.40 mmol, 70%). Yellow–brown solid; m.p.: 384.3 °C (decomp.); ^1^H NMR (200 MHz, DMSO-*d*_6_): *δ* = 7.68 (dd, *J* = 8.7, 1.9 Hz, 1H), 7.91 (d, *J* = 1.8 Hz, 1H), 8.16 (d, *J* = 8.7 Hz, 1H), 9.24 (s, 1H), 12.98 (s, 1H) ppm; ^13^C NMR (101 MHz, DMSO-*d*_6_): *δ* = 121.8, 126.6, 127.1, 128.2, 128.8, 131.4, 139.3, 143.0, 167.2 ppm.

#### 6-Chloro-3-nitroquinolin-4(1H)-one (5c)

[31] Applying general procedure D, 6-chloroquinolin-4(1*H*)-one (**4c**, 2.20 g, 12.70 mmol, 1.00 equiv.) was treated with conc. HNO_3_ (1.1 cm^3^) to give **5c** (1.60 g, 7.12 mmol, 56%). Cream-coloured solid; m.p.: > 300 °C (decomp.) (Ref. [31] > 300 °C (decomp.); ^1^H NMR (400 MHz, DMSO-*d*_6_): *δ* = 7.75 (d, *J* = 8.8 Hz, 1H), 7.85 (dd, *J* = 8.79, 2.45 Hz, 1H), 8.16 (d, *J* = 2.43 Hz, 1H), 9.24 (s, 1H), 13.15 (s, 1H) ppm; ^13^C NMR (101 MHz, DMSO-*d*_*6*_): *δ* = 121.9, 125, 129.4, 130.7, 131.1, 133.3, 137.1, 142.8, 166.6 ppm.

#### 6-Methoxy-3-nitroquinolin-4(1H)-one (5d)

[42] Applying general procedure D, 6-methoxyquinolin-4(1*H*)-one (**4d**, 2.20 g, 13.0 mmol, 1.00 equiv) was treated with conc. HNO_3_ (1.2 cm^3^) to give **5d** (2.10 g, 9.75 mmol, 75%). Yellow–brown solid; m.p.: 320 °C (decomp.) (Ref. [42] > 325 °C); ^1^H NMR (200 MHz, DMSO-*d*_6_): *δ* = 3.88 (s, 3H), 7.42 (dd, *J* = 9.0, 3.0 Hz, 1H), 7.59–7.75 (m, 2H), 9.12 (s, 1H), 12.99 (s, 1H) ppm; ^13^C NMR (101 MHz, DMSO-*d*_6_): *δ* = 55.6, 106.0, 121.2, 122.9, 129.5, 130.3, 132.6, 140.9, 157.4, 167.0 ppm.

#### 4-Chloro-3-nitroquinoline (6a)

[31] Applying general procedure E, 3-nitroquinolin-4(1*H*)-one (**5a**, 1.80 g, 9.46 mmol, 1.00 equiv) was treated with POCl_3_ (3.5 cm^3^) to give **6a** (1.83 g, 8.77 mmol, 93%). Pale yellow solid; m.p.: 119–120 °C (Ref. [31] 121–122 °C); ^13^C NMR (101 MHz, CDCl_3_): *δ* = 125.6, 126.1, 129.8, 130.4, 133.3, 136.7, 144.6, 149.3 ppm, signal of a quaternary carbon not detectable. ^1^H NMR spectral data were found to be identical to the ones described in Ref. [31].

#### 7-Bromo-4-chloro-3-nitroquinolin (6b)

[45] Applying general procedure E, 7-bromo-3-nitroquinolin-4(1*H*)-one (**5b**, 0.70 g, 2.60 mmol, 1.00 equiv.) was treated with POCl_3_ (1.0 cm^3^) to give **6b** (0.68 g, 2.37 mmol, 91%). Yellow–brown solid; m.p.: 150.9–151.1 °C; ^1^H NMR (400 MHz, CDCl_3_): *δ* = 7.90 (dd, *J* = 9.0, 1.9 Hz, 1H), 8.30 (d, *J* = 9.0 Hz, 1H), 8.41 (d, *J* = 1.9 Hz, 1H), 9.26 (s, 1H) ppm; ^13^C NMR (101 MHz, CDCl_3_): *δ* = 124.5, 127.3, 128.4, 132.8, 133.5, 137.0, 145.8, 149.7 ppm, signal of C3 not detectable.

#### 4,6-Dichloro-3-nitroquinoline (6c)

[31] Applying general procedure E, 6-chloro-3-nitroquinolin-4(1*H*)-one (**5c**, 0.50 g, 2.23 mmol, 1.00 equiv.) was treated with POCl_3_ (0.8 cm^3^) to **6c** (0.46 g, 1.89 mmol, 85%). Colorles solid; m.p.: 232–234 °C (Ref. [31] 165–167 °C); ^1^H NMR (400 MHz, CDCl_3_): *δ* = 7.88 (dd, *J* = 9.0, 2.3 Hz, 1H), 8.16 (dd, *J* = 9.0, 0.5 Hz, 1H,H8), 8.40 (dd, *J* = 2.3, 0.5 Hz, 1H), 9.23 (s, 1H) ppm; ^13^C NMR (101 MHz, CDCl_3_): *δ* = 124.9, 126.5, 132.0, 134.2, 135.6, 136.4, 141.8, 144.7, 147.7 ppm.

#### 4-Chloro-6-methoxy-3-nitroquinoline (6d)

[31] Applying general procedure E, 6-methoxy-3-nitroquinolin-4(1*H*)-one (**5d**, 2.00 g, 9.10 mmol, 1.00 equiv.) was treated with POCl_3_ (3.4 cm^3^) to give **6d** (0.90 g, 3.78 mmol, 42%). Yellow solid; m.p.: 113–114 °C (Ref. [31] 281–283 °C); ^1^H NMR (400 MHz, CDCl_3_): *δ* = 4.03 (s, 3H), 7.52–7.61 (m, 2H), 8.10 (dd, *J* = 8.7, 1.0 Hz, 1H), 9.09 (s, 1H) ppm; ^13^C NMR (151 MHz, CDCl_3_): *δ* = 56.1, 103.1, 126.3, 127.2, 131.9, 134.3, 141.9, 145.4, 160.5 ppm, signal of C3 not detectable.

#### 3-Nitroquinolin-4-amine (7a)

[44] Applying general procedure F, 4-chloro-3-nitroquinoline (**6a**, 1.00 g, 4.75 mmol, 1.00 equiv.) was treated with aq. NH_4_OH (25%, 50 cm^3^) to give **7a** (0.89 g, 4.70 mmol, quant.). Yellow solid; m.p.: 265–267 °C (Ref. [44] 265–266 °C); NMR spectral data were found to be identical to the ones described in Ref. [44].

#### 7-Bromo-3-nitroquinolin-4-amine (7b, C_9_H_6_BrN_3_O_2_)

 Appyling general procedure F, 7-bromo-4-chloro-3-nitroquinolin (**6b**, 0.679 g, 2.4 mmol, 1.00 equiv.) was treated with aq. NH_4_OH (25%, 24 cm^3^) to give **7b** (0.64 g, 2.30 mmol, quant.). Yellow solid; m.p.: 245–246 °C; ^1^H NMR (400 MHz, DMSO-*d*_6_): *δ* = 7.76 (dd, *J* = 8.9, 2.1 Hz, 1H), 8.05 (d, *J* = 2.1 Hz, 1H), 8.51 (d, *J* = 9.0 Hz, 1H), 9.05 (s, 2H), 9.15 (s, 1H) ppm; ^13^C NMR (101 MHz, DMSO-*d*_6_): *δ* = 117.8, 123.4, 126.4, 126.4, 129.2, 131.4, 148.2, 148.6, 149.1 ppm; HRMS: *m/z* calc. for C_9_H_7_BrN_3_O_2_ ([M + H]^+^) 267.9716, found 267.9724.

#### 6-Chloro-3-nitroquinolin-4-amine (7c, C_9_H_6_ClN_3_O_2_)

 Applying general procedure F, 4-chloro-3-nitroquinoline (**6c**, 0.17 g, 0.70 mmol, 1.00 equiv.) was treated with aq. NH_4_OH (25%, 7 cm^3^) to give **7c** (0.156 mg, 0.70 mmol, quant.). Yellow solid; m.p.: 295–297 °C; ^1^H NMR (400 MHz, DMSO-*d*_6_): *δ* = 7.85 (dd, *J* = 8.8, 2.0 Hz, 1H), 7.88 (dd, *J* = 8.9, 0.6 Hz, 1H), 8.76 (dd, *J* = 2.1, 0.7 Hz, 1H), 9.03 (s, 2H), 9.16 (s, 1H) ppm; ^13^C NMR (101 MHz, DMSO-*d*_6_): *δ* = 119.9, 123.5, 123.7, 131.1, 131.5, 132.9, 146.9, 147.4, 147.9 ppm; HRMS: *m/z* calc. for C_9_H_7_ClN_3_O_2_ ([M + H]^+^) 224.0221, found 224.0222.

#### 3,4-Diaminoquinoline (8a) 

[45] 3-Nitroquinolin-4-amine (**7a**, 850 mg, 4.50 mmol, 1.00 equiv.) was dissolved in methanol (250 cm^3^) and Pd/C (10% Pd, 85 mg) was added to the solution. Atmosphere was changed to H_2_ (balloon). The reaction was stopped after full consumption of starting material observed by TLC (CH_2_Cl_2_/MeOH, 19:1). The reaction mixture was filtered through a bed of Celite and solvent removed under reduced pressure to give **8a** (0.74 g, 4.28 mmol, 95%). Brown solid; m.p.: 167–169 °C (Ref. [45] 171–173 °C); ^1^H NMR (400 MHz, DMSO-*d*_6_): *δ* = 4.72 (br s, 2H), 5.87 (br s, 2H), 7.21–7.38 (m, 2H), 7.61–7.69 (m, 1H), 7.95–8.02 (m, 1H), 8.20 (s, 1H) ppm; ^13^C NMR (101 MHz, DMSO-*d*_6_): *δ* = 118.5, 121.0, 123.4, 124.4, 125.1, 128.6, 134.0, 141.0, 142.7 ppm.

#### 7-Bromoquinoline-3,4-diamine (8b, C_9_H_8_BrN_3_)

7-Bromo-3-nitroquinoline-4-amine (**7b**, 510 mg, 1.9 mmol, 1 equiv.) was suspended in EtOH/H_2_O (4:1, 20 cm^3^). Fe (1.05 g, 19 mmol, 10 equiv.) and NH_4_Cl (28.7 mg, 0.536 mmol, 1 equiv.) were then added to the mixture and the reaction was stirred and heated to reflux for up to 3 h until full consumption of starting material was observed by TLC (CH_2_Cl_2_/MeOH, 9:1, + 3% Et_3_N). The reaction was then filtered through a bed of Celite. The filtrate was concentrated on SiO_2_ and the product **8b** was obtained after purification by FCC using a gradient of MeOH in EtOAc + 3% Et_3_N (320 mg, 1.33 mmol 70%). Yellow–brown solid; m.p.: 126–128 °C; ^1^H NMR (400 MHz, DMSO-*d*_6_): *δ* = 4.84 (bs, 2H), 6.03 (s, 3H), 7.39 (dd, *J* = 9.0, 2.1 Hz, 1H), 7.81 (d, *J* = 2.1 Hz, 1H), 7.97 (d, *J* = 9.0 Hz, 1H), 8.19 (s, 1H) ppm; ^13^C NMR (101 MHz, DMSO-*d*_6_): *δ* = 117.2, 117.4, 123.4, 125.8, 126.2, 130.2, 134.3, 141.6, 143.4 ppm; HRMS: *m/z* calc. for C_9_H_9_BrN_3_ ([M + H]^+^) 237.9974, found 237.9983.

#### 6-Chloroquinoline-3,4-diamine (8c)

[17] In a screw cap vial, 6-chloro-3-nitroquinoline-4-amine (**7c**, 120 mg, 0.536 mmol, 1 equiv.) was suspended in EtOH/H_2_O (4:1, 5 cm^3^). Fe (300 mg, 5.36 mmol, 10 equiv.) and NH_4_Cl (28.7 mg, 0.536 mmol, 1 equiv.) were then added to the mixture and the reaction was stirred and heated to reflux for up to 3 h until full consumption of starting material was observed by TLC (CH_2_Cl_2_/MeOH, 9:1, + 3% Et_3_N). The reaction was then filtered through a bed of Celite. The filtrate was concentrated on SiO_2_ and the product **8c** was obtained after purification by FCC using a gradient of MeOH in EtOAc + 3% NEt_3_ (84 mg, 0.43 mmol, 80%). Brown solid; m.p.: 215–217 °C (Ref. [17] 206–209 °C); ^1^H NMR (400 MHz, DMSO-*d*_6_): *δ* = 4.89 (s, 2H), 5.94 (s, 2H), 7.28 (dd, *J* = 8.9, 2.3 Hz, 1H), 7.65 (d, *J* = 8.9 Hz, 1H), 8.11 (d, *J* = 2.3 Hz, 1H), 8.20 (s, 1H) ppm; ^13^C NMR (101 MHz, DMSO-*d*_6_): *δ* = 119.0, 120.1, 125.1, 126.1, 128.3, 130.3, 133.8, 140.4, 140.5 ppm.

#### 6-Methoxyquinoline-3,4-diamine (8d, C_10_H_11_N_3_O)

4-Chloro-6-methoxy-3-nitroquinoline (**6d**, 0.88 mg, 3.30 mmol, 1.00 equiv.) was dissolved in DMF (25 cm^3^). NaN_3_ (0.44 g, 6.60 mmol, 2.00 equiv.) was added and the reaction mixture was stirred for 20 min at rt. The solvent was removed in vacuo and the residue was dissolved in CH_2_Cl_2_. The organic layer was washed with water and brine and dried with MgSO_4_. The solvent was removed in vacuo and the product **7d** was obtained in good purity and quantitative yield. Due to the instability of the azide group it was immediately used for the next reaction step without further purification. 4-Azido-6-methoxy-3-nitroquinoline (**7d**, 0.84 g, 3.40 mmol, 1.00 equiv.) was dissolved in MeOH and after the addition of Pd/C (10% Pd, 84 mg) the reaction was set under a hydrogen atmosphere (1 bar) and stirred at rt for 24 h. The reaction mixture was filtered through a bed of Celite. The product **8d** was obtained after evaporation of the solvent (0.5 g, 2.54 mmol, 77%). Brown oil; ^1^H NMR (400 MHz, DMSO-*d*_6_): *δ* = 3.85 (s, 3H), 4.69 (s, 2H), 5.67 (s, 2H), 6.95 (dd, *J* = 9.1, 2.7 Hz, 1H), 7.30 (d, *J* = 2.8 Hz, 1H), 7.54 (d, *J* = 9.1 Hz, 1H), 8.06 (s, 1H) ppm; ^13^C NMR (101 MHz, CD_3_OD): *δ* = 56.0, 100.1, 119.8, 120.7, 125.4, 130.1, 137.9, 140.3, 140.5, 158.4 ppm; HRMS: *m/z* calc. for C_10_H_12_N_3_O ([M + H]^+^) 190.0982, found 190.0975.

#### Imidazo[4,5-*c*]quinoline (9a)

[45] 3,4-diaminoquinoline (**8a**, 1.47 g, 8.38 mmol, 1.00 equiv.) was suspended in trimethylorthoformate (170 cm^3^). The reaction mixture was heated under stirring until a clear solution was obtained. Then, formic acid (0.443 cm^3^, 11.8 mmol, 1.40 equiv.) was added carefully and a slight amount of precipitate was formed. Atmosphere was changed to argon, then reaction mixture was heated and stirred to reflux for 2 h, when full consumption of starting material was observed by TLC (CH_2_Cl_2_/MeOH, 9:1). The solvent was removed under reduced pressure and the solid obtained was washed several times with Et_2_O, sat. aq. NaHCO_3_, water and dried. The residual was purified by FCC (gradient of MeOH in CH_2_Cl_2_) to give the desired product **9a** (0.966 g, 6.29 mmol, 75%). Cream coloured powder; m.p.: 285–288 °C (Ref. [45] 285–288 °C); ^1^H NMR (400 MHz, DMSO-*d*_6_): *δ* = 7.62–7.73 (m, 2H), 8.07–8.15 (m, 1H), 8.35–8.42 (m, 1H), 8.48 (s, 1H), 9.21 (s, 1H) ppm; ^13^C NMR (101 MHz, DMSO-*d*_6_): *δ* = 119.2, 121.5, 126.2, 126.8, 129.5, 133.4, 137.0, 142.3, 142.4, 143.5 ppm.

#### 7-Bromo-5H-imidazo[4,5-*c*]quinoline (9b, C_10_H_6_BrN_3_)

7-Bromo-3,4-diaminoquinoline (**8b**, 20 mg, 0.08 mmol, 1 equiv.) was suspended in trimethylorthoformate (2 cm^3^). The reaction mixture was heated under stirring until a clear solution was obtained. Then, formic acid (4 mm^3^, 0.112 mmol, 1.4 equiv.) was added carefully and a slight amount of precipitate was formed. Atmosphere was changed to argon, then the reaction mixture was heated to reflux for 2 h when full consumption of starting material was observed by TLC (CH_2_Cl_2_/MeOH, 9:1). The solvent was removed under reduced pressure and the solid obtained was washed several times with Et_2_O, sat. aq. NaHCO_3_, water and dried in vacuo. The residual was purified by FCC (gradient of MeOH in CH_2_Cl_2_) to give the desired product **9b** (14 mg, 0.05 mmol, 57%). Colourless solid; m.p.: 312–313 °C; ^1^H NMR (600 MHz, DMSO-*d*_6_, mixture of tautomers): *δ* = 7.85 (d, *J* = 8.3 Hz, 1H), 8.32 (d, *J* = 2.0 Hz, 1H), 8.35 (d, *J* = 8.7 Hz, 1H), 8.55 (s, 1H), 9.25 (s, 1H), 13.40 (s, 1H) ppm; ^13^C NMR (151 MHz, DMSO-*d*_6_, mixture of tautomers): *δ* = 119.8, 123.6, 126.9, 128.2, 129.4, 131.4, 142.8, 143.9, 144.2 ppm; HRMS: *m/z* calc. for C_10_H_7_BrN_3_ ([M + H]^+^) 247.9818, found 247.9819.

#### 6-Chloro-3H-imidazo[4,5-*c*]quinoline (9c, C_10_H_6_ClN_3_)

6-Chloroquinoline-3,4-diamine (**8c**, 70 mg, 0.36 mmol, 1.00 equiv.) was suspended in trimethylorthoformate (7 cm^3^). The reaction mixture was heated under stirring until a clear solution was obtained. Formic acid (20 mm^3^, 0.5 mmol, 1.4 equiv.) was added carefully and a slight amount of precipitate was formed. Atmosphere was changed to argon and the reaction mixture was heated to reflux for 2 h until full consumption of starting material was observed by TLC (CH_2_Cl_2_/MeOH, 19:1). The solvent was removed under reduced pressure and the solid obtained was washed several times with Et_2_O, sat. aq. NaHCO_3_, water and dried in vacuo. The residual was purified by FCC (gradient of MeOH in CH_2_Cl_2_) to give the desired product **9c** (74 mg, 0.343 mmol, 95%). Cream coloured solid; m.p.: > 300 °C (decomp.); ^1^H NMR (400 MHz, DMSO-*d*_6_): *δ* = 7.69 (dd, *J* = 9.0, 2.4 Hz, 1H), 8.13 (d, *J* = 8.9 Hz, 1H), 8.44 (d, *J* = 2.5 Hz, 1H), 8.54 (s, 1H), 9.24 (s, 1H) ppm; ^13^C NMR (101 MHz, DMSO-*d*_6_): *δ* = 120.5, 127.4, 130.8, 131.7,141.9 ppm; HRMS: *m/z* calc. for C_10_H_7_ClN_3_ ([M + H]^+^) 204.0329, found 204.0334.

#### 8-Methoxy-5H-imidazo[4,5-*c*]quinoline (9d, C_11_H_9_N_3_O)

6-Methoxyquinoline-3,4-diamine (**8d**, 100 mg, 0.53 mmol, 1.00 equiv.) was suspended in trimethylorthoformate (11 cm^3^). The reaction mixture was heated under stirring until a clear solution was obtained. Then, formic acid (30 mm^3^, 0.74 mmol, 1.40 equiv.) was added carefully and slight amount of precipitate was formed. Atmosphere was changed to argon and the reaction mixture was heated to reflux for 2 h when full consumption of starting material was observed by TLC (CH_2_Cl_2_/MeOH, 19:1). The solvent was removed under reduced pressure and the solid obtained was washed several times with Et_2_O, sat. aq. NaHCO_3_, water and dried in vacuo. The residual was purified by FCC (gradient of MeOH in CH_2_Cl_2_) to give the desired product **9d** (50 mg, 0.25 mmol, 47%). Red solid; m.p.: 256–258 °C; ^1^H NMR (400 MHz, methanol-*d*_4_): *δ* = 3.99 (s, 3H), 7.32 (dd, *J* = 9.2, 2.8 Hz, 1H), 7.74 (s, 1H), 8.00 (d, *J* = 9.2 Hz, 1H), 8.41 (s, 1H), 8.97 (s, 1H) ppm; ^13^C NMR (101 MHz, methanol-*d*_4_): *δ* = 56.2, 101.4, 120.7, 131.1, 140.2, 143.6, 160.0 ppm, the signals of four carbons were not detectable; HRMS: *m/z* calc. for C_11_H_10_N_3_O ([M + H]^+^) 200.0818, found 200.0826.

#### 2-Phenylimidazo[4,5-*c*]quinoline (10a)

[45] (a) Starting from 3,4-diaminoquinoline (**8a**, 40 mg, 0.23 mmol, 1.00 equiv.) and benzaldehyde (25 mm^3^, 0.24 mmol, 1.05 equiv.). After the addition of PhNO_2_ (2.5 cm^3^), the reaction mixture was heated to 150 °C overnight, until full consumption of starting material was observed by TLC (CH_2_Cl_2_/MeOH, 9:1). Then, the mixture was concentrated on silica and the desired product **10a** was obtained by FCC using a gradient of MeOH in CH_2_Cl_2_ from 5 to 20% (56 mg, 0.23 mmol, 98%).

(b) Via CH activation: In a screw cap vial, imidazo[4,5-*c*]quinoline (**9a**, 60 mg, 0.35 mmol, 1.00 equiv.), Pd(OAc)_2_ (4 mg, 0.018 mmol, 5 mol%), and CuI (40 mg, 0.212 mmol, 0.6 equiv.) were placed. Atmosphere was changed to argon and degassed DMF (2 cm^3^) was added. Subsequently, iodobenzene (80 mm^3^, 0.71 mmol, 2.00 equiv.) was added via syringe. The vial was placed in a heating block and stirred at 140 °C for 48 h. Then, the reaction mixture was filtered through Celite and concentrated in vacuo. The desired product was isolated with preparative HPLC to yield **10a** (29 mg, 0.12 mmol, 34%). White-brown powder; m.p.: 283–285 °C (Ref. [45] 285–288 °C); ^1^H NMR (600 MHz, DMSO-*d*_6_): *δ* = 7.41 (t, *J* = 7.3 Hz, 1H), 7.50 (t, *J* = 7.6 Hz, 2H), 7.52–7.56 (m, 2H), 7.96–8.06 (m, 1H), 8.28–8.35 (m, 2H), 8.40–8.48 (m, 1H), 9.10 (s, 1H) ppm; ^13^C NMR (151 MHz, DMSO-*d*_6_): *δ* = 121.1, 122.7, 124.2, 125.4, 125.8, 127.1, 129.0, 129.7, 133.8, 138.2, 142.6, 142.9, 143.6, 156.5. ppm.

#### 8-Chloro-2-phenyl-3H-imidazo[4,5-*c*]quinoline (10c, C_16_H_10_ClN_3_)

6-Chloroquinoline-3,4-diamine (**9c**, 70 mg, 0.36 mmol, 1.00 equiv.) and benzaldehyde (39 mm^3^, 0.38 mmol, 1.05 equiv.). After the addition of PhNO_2_ (5 cm^3^), the reaction mixture was heated to 150 °C overnight, until full consumption of starting material was observed by TLC (CH_2_Cl_2_/MeOH, 19:1). The mixture was concentrated on silica and the desired product **10c** was obtained by FCC using a gradient of MeOH in CH_2_Cl_2_ from 5 to 20% (71 mg, 0,25 mmol, 70%). Brown powder; m.p.: > 300 °C (decomp.); ^1^H NMR (400 MHz, DMSO-*d*_6_): *δ* = 7.54–7.59 (m, 1H), 7.60–7.66 (m, 2H), 7.70 (dd, *J* = 8.9, 2.4 Hz, 1H), 8.13 (d, *J* = 8.9 Hz, 1H), 8.23–8.32 (m, 2H), 8.50–8.64 (m, 1H), 9.24 (s, 1H) ppm; ^13^C NMR (101 MHz, DMSO-*d*_6_): *δ* = 120.8, 126.8, 127.4, 129.2, 129.4, 130.5, 130.8, 131.8, 142.0, 152.3 ppm; HRMS: *m/z* calc. for C_16_H_11_ClN_3_ ([M + H]^+^) 280.0642, found 280.0632.

#### 4-(5H-Imidazo[4,5-*c*]quinolin-2-yl)benzonitrile (11a, C_17_H_10_N_4_)

3,4-Diaminoquinoline (**8a**, 80 mg, 0.5 mmol, 1.00 equiv.), 4-formylbenzonitrile (72 mg, 0.55 mmol, 1.1 equiv.) and ammonium acetate (39 mg, 0.5 mmol, 1.00 equiv.) were suspended in dry ethanol (5 cm^3^) and heated to reflux in an air atmosphere overnight until full consumption of starting material was observed by TLC (CH_2_Cl_2_/MeOH, 5:1), precipitation occurred and the solid was collected by filtration. The product **11a** was obtained by washing with EtOH and water and drying in vacuo (58 mg, 0.22 mmol, 43%). M.p.: 388–391 °C; ^1^H NMR (600 MHz, DMSO-*d*_*6*_): *δ* = 7.71–7.75 (m, 2H), 8.03–8.19 (m, 3H), 8.37–8.62 (m, 3H), 9.28 (s, 1H), 14.13 (s, 1H) ppm; ^13^C NMR (151 MHz, DMSO-*d*_*6*_): *δ* = 112.2, 117.3, 118.6, 121.5, 122.0, 126.6, 127.2, 127.4, 127.7, 128.8, 129.8, 133.2, 133.8, 135.4, 137.5, 138.0, 143.8, 144.7, 149.6, 150.4 ppm; HRMS: *m/z* calc. for C_17_H_11_N_4_ ([M + H]^+^) 271.0985, found 271.0993.

#### 4-(7-Bromo-5H-imidazo[4,5-*c*]quinolin-2-yl)benzonitrile (11b, C_17_H_9_BrN_4_)

7-Bromoquinoline-3,4-diamine (**8b**, 50 mg, 0.24 mmol, 1 equiv.) and 4-formylbenzonitrile (1.05 equiv.) were dissolved in PhNO_2_ (2.4 cm^3^), the reaction mixture was heated to 150 °C for 24 h, until full consumption of starting material was observed by TLC (CH_2_Cl_2_/MeOH, 5:1). Then, the reaction mixture was concentrated on silica and product **11b** were obtained by FCC, applying a gradient of MeOH in CH_2_Cl_2_ from 5 to 20% (52 mg, 0.17 mmol, 69%). Yellow solid; m.p.: 350 °C (decomp.); ^1^H NMR (400 MHz, DMSO-*d*_6_): *δ* = 7.82 (dd, *J* = 8.7, 2.0 Hz, 1H), 8.03 (d, *J* = 8.2 Hz, 2H), 8.26 (d, *J* = 1.9 Hz, 1H), 8.36 (d, *J* = 8.5 Hz, 3H), 9.22 (s, 1H) ppm; ^13^C NMR (101 MHz, DMSO-*d*_6_): *δ* = 112.7, 118.5, 119.0, 120.5, 124.3, 127.7, 129.9, 131.9, 133.5, 134.3, 139.2, 143.8, 144.8, 151.3 ppm; HRMS: *m/z* calc. for C_17_H_10_BrN_4_ ([M + H]^+^) 349.0083, found 349.0092.

#### 4-(8-Methoxy-5H-imidazo[4,5-*c*]quinolin-2-yl)benzonitrile (11d, C_18_H_12_N_4_O)

6-Methoxyquinoline-3,4-diamine (**8d**, 45 mg, 0.24 mmol, 1.00 equiv.) and 4-formylbenzonitrile (33 mg, 0.25 mmol, 1.05 equiv.) were dissolved in PhNO_2_ (2.4 cm^3^) and the reaction mixture was heated to 150 °C for 24 h, until full consumption of starting material was observed by TLC (CH_2_Cl_2_/MeOH, 5:1). Then, the reaction mixture was concentrated on silica and product **11d** was obtained by FCC, applying a gradient of MeOH in CH_2_Cl_2_ from 5 to 20% (37 mg, 0.12 mmol, 52%). Red solid; m.p.: 143–145 °C; ^1^H NMR (600 MHz, DMSO-*d*_6_): *δ* = 3.98 (s, 3H), 7.32 (dd, *J* = 9.1, 2.9 Hz, 1H), 7.84 (d, *J* = 2.8 Hz, 1H), 8.02 (d, *J* = 9.1 Hz, 1H), 8.08 (d, *J* = 8.5 Hz, 2H), 8.41 (d, *J* = 8.2 Hz, 2H), 9.09 (s, 1H) 13.93 (s, 1H) ppm; ^13^C NMR (151 MHz, DMSO-*d*_*6*_, mixture of tautomers): *δ* = 55.6, 100.8, 112.2, 118.6, 118.8, 123.8, 127.2, 129.3, 131.1, 133.1, 133.89, 139.2, 149.8, 157.6 ppm; HRMS: *m/z* calc. for C_18_H_13_N_4_O ([M + H]^+^) 301.1084, found 301.1088.

#### 7-Bromo-2-(4-methoxyphenyl)-5H-imidazo[4,5-*c*]quinoline (12b, C_17_H_12_BrN_3_O)

7-Bromoquinoline-3,4-diamine (**8b**, 50 mg, 0.29 mmol, 1 equiv.) and *p*-methoxybenzoic acid (53 mg, 0.35 mmol, 1.2 equiv.) were treated with PPA (0.5 cm^3^) was added and the reaction mixture was heated to 100 °C for 24 h, until full consumption of starting material was observed by TLC(CH_2_Cl_2_/MeOH, 5:1). The reaction mixture was then poured on H_2_O and adjusted to a pH of 9–10 with aq. NH_4_OH (25%), whereupon precipitation occurred. Precipitate was collected by filtration, washed with H_2_O and dried to give **12b** (57 mg, 0.22 mmol, 77%). Brown solid; m.p.: 108–110 °C; ^1^H NMR (400 MHz, DMSO-*d*_*6*_, mixture of tautomers): *δ* = 3.85 (s, 3H), 7.15 (d, *J* = 8.8 Hz, 2H), 7.80 (m, 1H), 8.19 (m, 2H), 8.27 (d, *J* = 2.0 Hz, 1H), 8.40 (d, *J* = 8.8 Hz, 1H), 9.15 or 9.21 (s, 1H), 13.62 or 13.75 (s, 1H) ppm; ^13^C NMR (101 MHz, DMSO-*d*_*6*_, mixture of tautomers): *δ* = 55.4, 113.7, 114.5, 119.6, 121.9, 123.7, 128.3, 129.1, 131.4, 137.8, 138.3, 144.2, 145.1, 152.0, 153.1, 161.0 ppm; HRMS: *m/z* calc. for C_17_H_13_BrN_3_O ([M + H]^+^) 354.0236, found 354.0237.

#### 8-Methoxy-2-(4-methoxyphenyl)-5H-imidazo[4,5-*c*]quinoline (12d, C_18_H_15_N_3_O_2_)

6-Methoxyquinoline-3,4-diamine (**8d**, 54 mg, 0.29 mmol, 1 equiv.) and *p*-methoxybenzoic acid (53 mg, 0.35 mmol, 1.2 equiv.) were treated with PPA (0.5 cm^3^) and the reaction mixture was heated to 100 °C for 24 h, until full consumption of starting material was observed by TLC (CH_2_Cl_2_/MeOH, 5:1). The reaction mixture was then poured on H_2_O and adjusted to a pH of 9–10 with aq. NH_4_OH (25%), whereupon precipitation occurred. Precipitate was collected by filtration, washed with H_2_O and dried to yield the desired product **12d** (24 mg, 0.08 mmol, 28%). Red oil; ^1^H NMR (600 MHz, DMSO-*d*_6_): *δ* = 3.86 (s, 3H), 3.98 (s, 3H), 7.17 (d, *J* = 8.8 Hz, 2H), 7.29 (dd, *J* = 9.1, 2.9 Hz, 1H), 7.82–7.87 (m, 1H), 7.99 (d, *J* = 9.1 Hz, 1H), 8.21 (d, *J* = 8.3 Hz, 2H), 9.02 (s, 1H), 13.55 (s, 1H) ppm; ^13^C NMR (151 MHz, DMSO-*d*_6_): *δ* = 55.4, 55.6, 100.7, 114.5, 118.4, 122.2, 128.3, 131.1, 134.7, 137.7, 139.2, 141.4, 151.6, 157.3, 160.9 ppm; HRMS: *m/z* calc. for C_18_H_16_N_3_O_2_ ([M + H]^+^) 306.1237, found 306.1231.

### Supplementary Information

Below is the link to the electronic supplementary material.

Supplementary file1 (DOCX 11588 KB)
