# Supplementary material for: Novel synthetic procedures for C2 substituted imidazoquinolines as ligands for the α/β-interface of the GABAA-receptor
Source: Monatsh Chem. 2022 Oct 29;154(12):1391–404. doi: 10.1007/s00706-022-02988-8 (PMC10667396; doi:10.1007/s00706-022-02988-8)
Supplement: Supplementary file 1 — Supplementary file1 (DOCX 11588 KB) [file 706_2022_2988_MOESM1_ESM.docx]

**Supporting Information**

Novel synthetic procedures for C2 substituted imidazoquinolines as ligands for the α/β-interface of the GABA_A_-receptor

Markus Draskovits,^a^ Daniele Catorci,^a^ Laurin Wimmer,^a^ Sabah Rehman,^b^ David Chan Bodin Siebert,^a^ Margot Ernst,^b^ Michael Schnürch*,^a^ Marko D. Mihovilovic,^a^

^a^ Institute of Applied Synthetic Chemistry, TU Wien, Getreidemarkt 9/163, 1060 Vienna, Austria, Email: michael.schnuerch@tuwien.ac.at

^b^ Center for Brain Research, Medical University of Vienna, Spitalgasse 4, A-1090 Vienna, Austria

Contents

[NMR Spectra of new and final compounds 3](#_Toc98489749)

[7-Bromo-3-nitroquinolin-4-amine (7b) 3](#_Toc98489750)

[7-Bromoquinoline-3,4-diamine (8b) 4](#_Toc98489751)

[6-Methoxyquinoline-3,4-diamine (8d) 5](#_Toc98489752)

[Imidazo[4,5-c]quinoline (9a) 6](#_Toc98489753)

[7-Bromo-5H-imidazo[4,5*-c*]quinoline (9b) 7](#_Toc98489754)

[6-Chloro-3*H*-imidazo[4,5-*c*]quinoline (9c) 8](#_Toc98489755)

[8-Methoxy-5H-imidazo[4,5*-c*]quinoline (9d) 9](#_Toc98489756)

[2-Phenyl-imidazo[4,5-c]quinoline (10a): 10](#_Toc98489757)

[8-Chloro-2-phenyl-3*H*-imidazo[4,5-*c*]quinoline (10c) 11](#_Toc98489758)

[7-Bromo-2-(4-methoxyphenyl)-5H-imidazo[4,5*-c*]quinoline (12b) 12](#_Toc98489759)

[8-Methoxy-2-(4-methoxyphenyl)-5H-imidazo[4,5*-c*]quinoline (12d) 13](#_Toc98489760)

[4-(5H-Imidazo[4,5-*c*]quinolin-2-yl)benzonitrile (11a) 14](#_Toc98489761)

[4-(7-Bromo-5H-imidazo[4,5*-c*]quinolin-2-yl)benzonitrile (11b) 15](#_Toc98489762)

[4-(8-Methoxy-5H-imidazo[4,5*-c*]quinolin-2-yl)benzonitrile (11d) 16](#_Toc98489763)

[CH-activation of N-protected imidazoquinolines 17](#_Toc98489764)

[Synthesis of N-protected imidazoquinolines 17](#_Toc98489765)

[CHA of partially protected imidazoquinolines 17](#_Toc98489766)

[SI Table 1: Reaction conditions optimization of CH-activation 19](#_Toc98489767)

[Tert-butyl-3H-imidazo[4,5-*c*]quinoline-3-carboxylate (13) 20](#_Toc98489768)

[Tert-butyl-1H-imidazo[4,5-*c*]quinoline-3-carboxylate (14) 21](#_Toc98489769)

[3-((2-(Trimethylsilyl)ethoxy)methyl)-3H-imidazo[4,5-c]quinoline (15) 22](#_Toc98489770)

[3-((2-(Trimethylsilyl)ethoxy)methyl)-1H-imidazo[4,5-c]quinoline (16) 23](#_Toc98489771)

[2-Phenyl-3-((2-(trimethylsilyl)ethoxy)methyl)-3*H*-imidazo[4,5-c]quinoline (18) 24](#_Toc98489772)

## NMR Spectra of new and final compounds

7-Bromo-3-nitroquinolin-4-amine (7b)

7-Bromoquinoline-3,4-diamine (8b)

6-Methoxyquinoline-3,4-diamine (8d)

Imidazo[4,5-c]quinoline (9a)

7-Bromo-5H-imidazo[4,5*-c*]quinoline (9b)

6-Chloro-3*H*-imidazo[4,5-*c*]quinoline (9c)

8-Methoxy-5H-imidazo[4,5*-c*]quinoline (9d)

2-Phenyl-imidazo[4,5-c]quinoline (10a):

8-Chloro-2-phenyl-3*H*-imidazo[4,5-*c*]quinoline (10c)

7-Bromo-2-(4-methoxyphenyl)-5H-imidazo[4,5*-c*]quinoline (12b)

8-Methoxy-2-(4-methoxyphenyl)-5H-imidazo[4,5*-c*]quinoline (12d)

4-(5H-Imidazo[4,5-*c*]quinolin-2-yl)benzonitrile (11a)

4-(7-Bromo-5H-imidazo[4,5*-c*]quinolin-2-yl)benzonitrile (11b)

4-(8-Methoxy-5H-imidazo[4,5*-c*]quinolin-2-yl)benzonitrile (11d)

# CH-activation of N-protected imidazoquinolines

## Synthesis of N-protected imidazoquinolines

Tert-butyl-3H-imidazo[4,5-c]quinoline-3-carboxylate (**21**) and tert-butyl-1H-imidazo[4,5-c]quinoline-3-carboxylate (**22**): In a screw cap vial, imidazo[4,5-c]quinoline **17a** (63 mg, 0.37 mmol, 1.00 equiv.) was suspended in THF (2 mL). Di-tertbutyldicarbonate (162 mg, 0.744 mmol, 2.00 equiv.) and NEt_3_ (100 μL, d = 0.73 g/cm3, 0.744 mmol, 2.00 equiv.) were added. The resulting reaction mixture was stirred at rt for up to 24 h until full consumption of starting material was observed by TLC. The solvent was removed in vacuo and the residue obtained was dissolved in EtOAc (6 mL). The organic layer was washed with water (1x) and brine (1x), dried over NaSO_4_, filtered and evaporated to obtain a pale yellow solid. The regioisomers **21** and **22** were isolated by FCC (LP/EtOAc 3:1) with 78% and 6% yields, respectively.

Tert-butyl-3H-imidazo[4,5-*c*]quinoline-3-carboxylate (**21**): colourless solid, m.p. 134-136 °C, TLC: R_f_ 0.58 (LP/EtOAc 1:1), ^1^H-NMR (400 MHz, DMSO-*d*_6_) δ 1.71 (s, 9H), 7.72 – 7.85 (m, 2H), 8.12 – 8.19 (m, 1H), 8.43 – 8.50 (m, 1H), 8.93 (s, 1H), 9.45 (s, 1H), ^13^C NMR (101 MHz, DMSO-*d*_6_) δ 27.5, 86.7, 121.4, 121.7, 124.8, 127.5, 128.3, 129.4, 138.6, 144.2, 144.4, 144.7, 147.0 ppm. HRMS calc. for C_15_H_16_N_3_O_2_ [M + H]+ 270.1243, found 270.1237.

Tert-butyl-1H-imidazo[4,5-*c*]quinoline-3-carboxylate (**22**): brown solid, m.p. 140-142 °C, Rf = 0.23 (1:1 PE/EtOAc), 1H-NMR (400 MHz, DMSO-*d*_6_) δ 1.70 (s, 9H), 7.72 (ddd, *J* = 8.4, 6.9, 1.5 Hz, 1H), 7.79 (ddd, *J* = 8.4, 6.9, 1.6 Hz, 1H), 8.16 – 8.21 (m, 1H), 8.88 (s, 1H), 9.08 (ddd, *J* = 8.4, 1.6, 0.6 Hz, 1H), 9.31 (s, 1H) ppm. ^13^C-NMR (101 MHz, DMSO-*d*_6_) δ 27.5, 87.0, 117.4, 124.9, 126.5, 128.2, 130.1, 131.8, 137.8, 144.9, 145.7, 147.5 ppm HR-MS calc. for C_15_H_16_N_3_O_2_ [M + H]^+^ 270.1243, found 270.1237.

3-((2-(trimethylsilyl)ethoxy)methyl)-3H-imidazo[4,5-c]quinoline (**23**) and 3-((2-(trimethylsilyl)ethoxy)methyl)-1H-imidazo[4,5-c]quinoline (**24**): In a screw cap vial, 60 mg of **17a** (0.354 mmol, 1 equiv.) were dissolved in dry DMF (1.5 mL). DIPEA (155 μL, 0.886 mmol, 2.5 equiv.) and 2-(trimethylsilyl)ethoxymethyl chloride (70 μL, 0.389 mmol, 1.1 equiv.) were added slowly to the solution under a stream of argon. The resulting reaction mixture was heated and stirred to 80 °C for 4 h. The reaction was then quenched with water and extracted with EtOAc (3x). The organic layers were collected and washed with water (1x) and brine (1x), dried over NaSO4 and filtered. The filtrate was evaporated to give a brown oil. The regioisomers **23** and **24** were isolated by FC 5% MeOH in EtOAc with an overall 98% yield (70% and 28% yields of **23** and **24**, respectively).

3-((2-(trimethylsilyl)ethoxy)methyl)-3H-imidazo[4,5-c]quinoline (**23**): Rf = 0.59 (10% MeOH in CH_2_Cl_2_), ^1^H-NMR (400 MHz, CDCl_3_) δ -0.05 (s, 9H), 0.85 – 0.98 (m, 2H), 3.51 – 3.64 (m, 2H), 5.72 (s, 2H), 7.64 – 7.77 (m, 2H), 8.16 (s, 1H), 8.20 – 8.27 (m, 1H), 8.52 – 8.64 (m, 1H), 9.25 (s, 1H), ^13^C NMR (151 MHz, CDCl_3_) δ -1.3, 17.9, 67.2, 75.2, 121.9, 122.6, 127.1, 127.3, 127.8, 129.7, 136.4, 143.5, 144.6, 145.6 ppm, HR-MS calc for C_16_H_22_N_3_OSi [M + H]^+^ 300.1533, found 300.1527.

3-((2-(trimethylsilyl)ethoxy)methyl)-1H-imidazo[4,5-c]quinoline (**24**): Rf = 0.21 (10% MeOH in CH_2_Cl_2_), ^1^H NMR (400 MHz, CDCl_3_) δ = -0.07 (s, 9H), 0.89 – 0.99 (m, 2H), 3.56 – 3.66 (m, 2H), 5.85 (s, 2H), 7.66 (ddd, *J* = 8.3, 7.0, 1.4 Hz, 1H), 7.72 (ddd, *J* = 8.4, 7.0, 1.5 Hz, 1H), 8.04 (s, 1H), 8.26 – 8.31 (m, 1H), 8.40 – 8.46 (m, 1H), 9.36 (s, 1H), 13C NMR (151 MHz, CDCl3) δ -1.3, 17.8, 66.8, 76.1, 117.9, 122.1, 127.0, 127.8, 130.5, 133.5, 138.5, 143.5, 145.0, 145.6 ppm. HR-MS calc. for C_16_H_22_N_3_OSi [M + H]^+^ 300.1533, found 300.1527.

## CHA of partially protected imidazoquinolines

In a screw cap vial, Boc-protected imidazoquinoline **21** (60 mg, 0.22 mmol, 1.00 equiv.), Pd(OAc)_2_ (2.5 mg, 0.011 mmol, 5 mol%), CuI (4.3 mg, 0.022 mmol, 10 mol%) and Cs_2_CO_3_ (145 mg, 0.46 mmol, 2.00 equiv.) were weighted in. The vial was evacuated and backfilled with Argon (3x) and degassed DMF (2 mL) was added. Iodobenzene (50 µL, 0.71 mmol, 2.00 equiv.) was then added to the mixture. The reaction was heated to 140 °C for 48h. After this time, only deprotected imidazoquinoline 17a was isolated.

In a screw cap vial, protected imidazoquinoline **23** (60 mg, 0.22 mmol, 1.00 equiv.), Pd(OAc)_2_ (2.5 mg, 0.011 mmol, 5 mol%), CuI (4.3 mg, 0.022 mmol, 10 mol%) and Cs_2_CO_3_ (145 mg, 0.46 mmol, 2.00 equiv.) were weighted in. The vial was evacuated and backfilled with Argon (3x) and degassed DMF (2 mL) was added. Iodobenzene (50 µL, 0.71 mmol, 2.00 equiv.) was then added to the mixture. The reaction was heated to 140 °C for 48h. After this time, the reaction mixture was filtered through Celite and the concentrated. The product **25** was isolated with preparative HPLC (10 mg, 0.027 mmol, 12%): brown oil, R_f_ = 0.59 (10% MeOH in CH_2_Cl_2_), ^1^H-NMR (400 MHz, DMSO) δ -0.11 (s, 9H), 0.82 – 0.88 (m, 2H), 3.58 – 3.67 (m, 2H), 5.87 (s, 2H), 7.64 (tt, J = 3.8, 2.7 Hz, 3H), 7.70 –7.76 (m, 2H), 8.02 – 8.06 (m, 2H), 8.15 – 8.18 (m, 1H), 8.50 – 8.55 (m, 1H), 9.46 (s, 1H) ppm. 13C NMR (151 MHz, DMSO) δ -0.5, 18.1, 67.1, 74.4, 122.4, 122.5, 127.4, 127.8, 128.3, 129.6,129.9, 130.1, 130.3, 130.5, 130.6, 131.5, 138.3, 143.7, 145.0, 155.4 ppm. HRMS calc. for C_22_H_26_N_3_OSi [M + H]^+^ 376.1800, found 376.1840.

# SI Table 1: Reaction conditions optimization of CH-activation

| entry | Ar-X | (equiv.) | Catalyst |  | Base | Equiv. | Co-Catalyst/ ligands |  | solvent | T [°C] | outcome |
| --- | --- | --- | --- | --- | --- | --- | --- | --- | --- | --- | --- |
| 1 | Ar-I | 2 | Pd(OAc)_2_ | 5 mol% | - |  | CuI |  | DMF | 120 | Product formation of 10% |
| 2 | Ar-I | 2 | Pd(OAc)_2_ | 5 mol% | - |  | - |  | DMF | 120 | Increased side product formation |
| 3 | Ar-Cl | 1.5 | Ni(OTf)_2_ | 10 mol% | K_3_PO_4_ | 3 | dcype | 12 mol% | t-amylOH | 120 | No product formation |
| 4 | Ar-I | 2 | Pd(OAc)_2_ | 5 mol% | K_2_CO_3_ | 2 | P(2-furyl)_3_ | 10 mol% | DMF | 120 | Formation of product with side products |
| 5 | Ar-I | 2 | [RhCl(cod)_2_]_2_ | 5 mol% | DIPEA | 3 | P(cy)_3_ | 30 mol% | ODCB | 250 |  |
| 6 | Ar-Br | 2.5 | [RhCl_2_(p-cymene)]_2_ | 5 mol% | K_2_CO_3_ | 2.5 | Ph_2_CHCO_2_H | 30 mol% | NMP | 120 | Formation of product in a low yield |
| 7 | Ar-Br | 2.5 | [RhCl_2_(p-cymene)]_2_ | 5 mol% | K_2_CO_3_ | 2.5 | Ph_2_CHCO_2_H | 30 mol% | DMF | 120 | Formation of product in a low yield |
| 8 | Ar-Br | 2.5 | [RhCl_2_(p-cymene)]_2_ | 5 mol% | K_2_CO_3_ | 2.5 | PPh_3_ | 30 mol% | NMP | 120 | Formation of product in low yield, decreased side product formation |
| 9 | Ar-Br | 2.5 | [RhCl_2_(p-cymene)]_2_ | 5 mol% | K_2_CO_3_ | 2.5 | 1-AlCO_2_H | 30 mol% | NMP | 120 | Formation of product in low yield, decreased side product formation |
| 10 | Ar-I | 2.5 | [RhCl_2_(p-cymene)]_2_ | 5 mol% | K_2_CO_3_ | 2.5 | Ph_2_CHCO_2_H | 30 mol% | NMP | 120 | Less product formation than Ar-Br |
| 11 | Ar-Br | 2.5 | [RhCl_2_(p-cymene)]_2_ | 5 mol% | Cs_2_CO_3_ | 2.5 | Ph_2_CHCO_2_H | 30 mol% | NMP | 120 | Very poor conversion |
| 12 | Ar-Br | 2.5 | [RhCl_2_(benzene)]_2_ | 5 mol% | K_2_CO_3_ | 2.5 | Ph_2_CHCO_2_H | 30 mol% | NMP | 120 | No product formation |
| 13 | Ar-Br | 2.5 | [RhCl(cod)_2_]_2_ | 5 mol% | K_2_CO_3_ | 2.5 | Ph_2_CHCO_2_H | 30 mol% | NMP | 120 | No product formation |
| 14 | Ar-Br | 2.5 | [RhCl_2_(p-cymene)]_2_ | 5 mol% | K_2_CO_3_ | 2.5 | PPh_3_ | 30 mol% | NMP | 200 | Product and side product formation in low selectivity |
| 15 | Ar-I | 2 | Pd(OAc)_2_ | 5 mol% | K_2_CO_3_ | 2.5 | P(cy)_3_ | 30 mol% | NMP | 200 | Greater product formation with decreased side product formation |

Tert-butyl-3H-imidazo[4,5-*c*]quinoline-3-carboxylate (13)

Tert-butyl-1H-imidazo[4,5-*c*]quinoline-3-carboxylate (14)

3-((2-(Trimethylsilyl)ethoxy)methyl)-3H-imidazo[4,5-c]quinoline (15)

3-((2-(Trimethylsilyl)ethoxy)methyl)-1H-imidazo[4,5-c]quinoline (16)

2-Phenyl-3-((2-(trimethylsilyl)ethoxy)methyl)-3*H*-imidazo[4,5-c]quinoline (18)
